# Amide proton transfer-weighted imaging and stretch-exponential model DWI based ^18^F-FDG PET/MRI for differentiation of benign and malignant solitary pulmonary lesions

**DOI:** 10.1186/s40644-024-00677-9

**Published:** 2024-03-04

**Authors:** Nan Meng, Chen Song, Jing Sun, Xue Liu, Lei Shen, Yihang Zhou, Bo Dai, Xuan Yu, Yaping Wu, Jianmin Yuan, Yang Yang, Zhe Wang, Meiyun Wang

**Affiliations:** 1 Department of Medical Imaging, Henan Provincial People’s Hospital & Zhengzhou University People’s Hospital, Zhengzhou, China; 2 Laboratory of Brain Science and Brain-Like Intelligence Technology, Institute for Integrated Medical Science and Engineering, Henan Academy of Sciences, Zhengzhou, China; 3 Biomedical Research Institute, Henan Academy of Sciences, Zhengzhou, China; 4 Hematology Laboratory, the First Affiliated Hospital of Xinxiang Medical University, Xinxiang, China; 5 Department of Pediatrics, Zhengzhou Central Hospital Affiliated to Zhengzhou University & Zhengzhou Central Hospital, Zhengzhou, China; 6 Department of Medical Imaging, Xinxiang Medical University People’s Hospital & Henan Provincial People’s Hospital, Zhengzhou, China; 7 Central Research Institute, United Imaging Healthcare Group, Shanghai, China; 8 Beijing United Imaging Research Institute of Intelligent Imaging, United Imaging Healthcare Group, Beijing, China

**Keywords:** Lung diseases, Diffusion magnetic resonance imaging, Positron-emission tomography, Fluorodeoxyglucose F18

## Abstract

**Objectives:**

To differentiate benign and malignant solitary pulmonary lesions (SPLs) by amide proton transfer-weighted imaging (APTWI), mono-exponential model DWI (MEM-DWI), stretched exponential model DWI (SEM-DWI), and ^18^F-FDG PET-derived parameters.

**Methods:**

A total of 120 SPLs patients underwent chest ^18^F-FDG PET/MRI were enrolled, including 84 in the training set (28 benign and 56 malignant) and 36 in the test set (13 benign and 23 malignant). MTRasym(3.5 ppm), ADC, DDC, α, SUV_max_, MTV, and TLG were compared. The area under receiver-operator characteristic curve (AUC) was used to assess diagnostic efficacy. The Logistic regression analysis was used to identify independent predictors and establish prediction model.

**Results:**

SUV_max_, MTV, TLG, α, and MTRasym(3.5 ppm) values were significantly lower and ADC, DDC values were significantly higher in benign SPLs than malignant SPLs (all *P* < 0.01). SUV_max_, ADC, and MTRasym(3.5 ppm) were independent predictors. Within the training set, the prediction model based on these independent predictors demonstrated optimal diagnostic efficacy (AUC, 0.976; sensitivity, 94.64%; specificity, 92.86%), surpassing any single parameter with statistical significance. Similarly, within the test set, the prediction model exhibited optimal diagnostic efficacy. The calibration curves and DCA revealed that the prediction model not only had good consistency but was also able to provide a significant benefit to the related patients, both in the training and test sets.

**Conclusion:**

The SUV_max_, ADC, and MTRasym(3.5 ppm) were independent predictors for differentiation of benign and malignant SPLs, and the prediction model based on them had an optimal diagnostic efficacy.

## Introduction

Solitary pulmonary lesions (SPLs) encompass single well-defined solid or sub-solid lung lesions surrounded by normal lung tissue and lack signs such as atelectasis or significant pleural effusion [1, 2]. In recent years, due to increasing environmental pollution, the prevalence of tobacco usage, and the increased awareness of health check-ups, the detection of SPLs has been on the rise [3]. Although most SPLs are eventually determined to be benign, early differentiation between benign and malignant lesions remains crucial for effective patient management [4]. For instance, accurate identification and prompt resection of malignant SPLs can improve the 5-year survival rate of patients with non-small cell lung cancer [5]. Similarly, accurately identifying benign SPLs before treatment could avoid unnecessary interventions, optimise the allocation of healthcare resources, and alleviate patient suffering [6]. Needle biopsy is currently an accepted method for distinguishing between benign and malignant SPLs prior to treatment. However, its limitations, such as small sample size and invasiveness, not only hinder its ability to accurately represent the characteristics of SPLs but also pose challenges for patients in poor physical condition or with lesions near vital structures such as the heart or large blood vessels [7, 8]. Therefore, finding a non-invasive method that can differentiate between benign and malignant SPLs prior to treatment holds importance for the patients involved.

In clinical practice, computed tomography (CT) is commonly used as the primary modality for evaluating patients with SPLs. However, it has inherent limitations, including exposure to ionising radiation and reliance solely on morphological criteria [4]. One of the most valuable diagnostic and evaluation tools for oncology is ^18^Fluorine-fluorodeoxyglucose positron emission tomography/magnetic resonance imaging (^18^F-FDG PET/MRI). This imaging technique not only provides information about glucose metabolism through ^18^F-FDG PET imaging but also enables the simultaneous acquisition of multiple quantitative MRI sequences during PET imaging, offering a more comprehensive assessment for clinical decision-making in these patients [9]. Amide proton transfer-weighted imaging (APTWI), mono-exponential model diffusion-weighted imaging (MEM-DWI), and stretched exponential model DWI (SEM-DWI) are quantitative MRI imaging sequences. APTWI allows the evaluation of mobile protein and peptide content in biological tissues without using exogenous contrast agents [10], while MEM-DWI and SEM-DWI provide insights into water molecule diffusion and tissue heterogeneity within the body [11]. Currently, ^18^F-FDG PET and MEM-DWI have shown promising results in differentiating between benign and malignant lung lesions and are widely used [12]. However, APTWI and SEM-DWI are still in the early stages of research regarding the differentiation between benign and malignant lung lesions, with limited studies available, often characterised by small sample sizes and the exclusion of SEM-DWI [13, 14]. Moreover, to the best of our knowledge, no systematic comparison has been made to assess the diagnostic performance of ^18^F-FDG PET, APTWI, MEM-DWI, and SEM-DWI in differentiating malignant from benign SPLs, and there is a lack of guidance on which parameters should be selected to help establish a clinical diagnosis.

This study aimed to use a hybrid ^18^F-FDG PET/MRI scanner to perform simultaneous chest ^18^F-FDG PET, APTWI, MEM-DWI, and SEM-DWI scans in patients with SPLs to compare the differences in each quantitative/semi-quantitative parameter between the benign and malignant groups, identify independent predictors, and establish a prediction model and validate it. The ultimate goal is to offer a novel reference for the clinical management of these patients.

## Materials and methods

### Patients

**Fig. 1:**
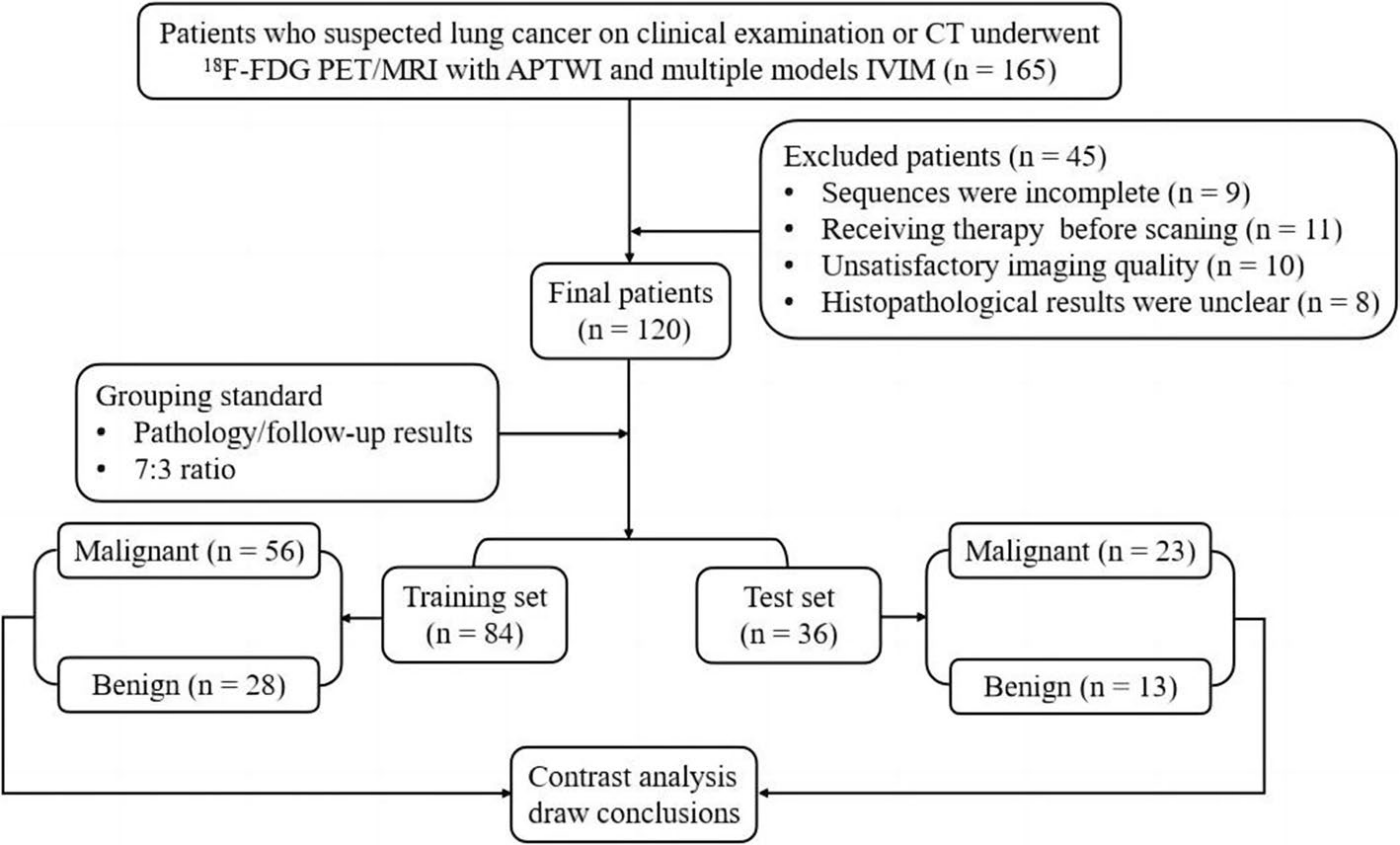
Flow diagram of the patient selection process

This study was approved by the ethics review committee at our hospital, and written informed consent was obtained from all patients. From August 2020 to April 2022, a series of 165 patients diagnosed with SPLs on CT underwent chest ^18^F-FDG PET/MRI. The following patients were excluded from the analysis: (i) those unable to complete all imaging sequences due to claustrophobia or other physical symptoms (*n* = 9); (ii) patients who had previously undergone radiotherapy, chemotherapy, or surgery prior to the ^18^F-FDG PET/MRI scan (*n* = 11); (iii) patients with poor image quality for ^18^F-FDG PET, APTWI, MEM-DWI, or SEM-DWI, making analysis challenging (*n* = 10); (iv) patients with missing clinical or histopathological information (*n* = 15). A total of 120 patients with SPLs were enrolled, and information on age, sex, smoking history, and maximum diameter of the lesion was collected. The study flow is presented in Fig. 1.

### Image acquisition

A chest scan was conducted using a hybrid 3.0 T PET/MRI system (uPMR 790, United Imaging, Shanghai, China) equipped with a 12-channel phased-array body coil. The ^18^F-FDG used in this study was produced by FracerLab FX-FDG (GE Minitrac) with a purity of > 95% and a pH of 4.5–8.5. All patients fasted for a minimum of 6 h before the scan to ensure that their serum glucose levels were < 6.5 mmol/L while injecting ^18^F-FDG (0.11 mCi/kg). The PET scan began 60 min after administering ^18^F-FDG and lasted for 27 min. All patients were placed in the supine position headfirst and were scanned from the upper thoracic inlet to the lower lung margin. All patients underwent breathing training before scanning in order to maintain smooth breathing during scanning and reduce image artefacts. A breathing strap was attached to monitor respiration by attaching it to the patient’s abdomen. Magnetic resonance-based attenuation correction was performed using a three-dimensional T1-weighted spoiled gradient-echo sequence with Dixon-based water-fat separation imaging. This technique enabled the segmentation of corrected images into soft tissue, fat, lung, and air compartments [15, 16]. The PET images were reconstructed using ordered subsets expectation maximisation with two iterations, 20 subsets, and a voxel size of 2.6 × 2.6 × 2.0 mm^3^. Simultaneously, during the ^18^F-FDG PET scan, axial T1-weighted imaging, T2-weighted imaging (T2WI), SEM-DWI, and APTWI were sequentially performed. The APTWI parameters were as follows: B_1_ values of 1.3 μT and 2.5 μT, ETL of 39, Gaussian pulse, 10 repeats, 100 ms duration, with an additional S_0_ image without chemical exchange saturation transfer (CEST) saturation pulse for normalisation, and Δ ranging from -4.5 to 4.5 ppm in 31 steps. Additionally, 11 low-power B_1_ images (B_1_ = 0.13 μT) were acquired with Δ ranging from -1.0 to 1.0 ppm for wide-angle staring synthetic aperture radar images used in B_0_ map correction. Table 1 provides a comprehensive description of the protocol details.

**Table 1:** Details of scanning protocol

Sequence	Wfi3d-trig	T1WI	T2WI	SEM-DWI	APTWI
Type / Orientation	FSE /Axial	FSE /Axial	FSE /Axial	SS—EPI /Axial	FSE /Axial
Field of view (cm^2^)	35 × 50	35 × 50	35 × 50	35 × 50	35 × 50
Repetition time (ms)	4.92	5.06	3315	1620	4500
Echo time (ms)	2.24	2.1	87.8	69.6	42.56
Matrix	192 × 192	303 × 456	264 × 480	202 × 256	128 × 100
Slice thickness (mm)	2	5	5	5	5
Interval (mm)	0	1	1	1	1
Number of excitations	2	2	2	1, 1, 2, 2, 4, 4, 6, 6, 8, 10	1
b-values (s/mm^2^)	/	/	/	0, 25, 50, 100, 150, 200, 400, 600, 800, 1000	/
Fat suppression	No	No	Yes	Yes	No
Respiratory compensation	Yes	Yes	Yes	Yes	Yes
Scan time	2 min 04 s	14 s	2 min 26 s	3 min 38 s	3 min 15 s (single slice)

*Wfi3d-trig* 3D T1-weighted spoiled gradient-echo sequence with Dixon-based water-fat separation imaging, *FSE* Fast spin echo, *SS-EPI* Single Shot Echo Planar Imaging, *T1WI* T1-weighted imaging, *T2WI* T2-weighted imaging, *SEM-DWI* Stretch-exponential model diffusion-weighted imaging, *APTWI* Amide proton transfer-weighted imaging

### Parameter generation

All images were uploaded to the post-processing Workstation (uWS-MR005, United Imaging, Shanghai, China) for motion correction and analysis. Fused PET/MRI software was used to automatically extract the volume of interest (VOI), determine the maximum standardised uptake value (SUV_max_), and quantify the metabolic tumour volume (MTV) and total lesion glycolysis (TLG) using a 40% SUV_max_ threshold [17]. Advanced analysis toolkit software, specifically diffusion analysis and CEST software, were used to process the MEM-DWI, SWM-DWI, and APTWI data. The parameters for MEM-DWI and SEM-DWI were calculated using Eqs. 1 and 2:

1 $${\mathrm S}_{\mathrm b}={\mathrm S}_0\,\exp\left(-\mathrm b\cdot\mathrm{ADC}\right)$$

2 $${S}_{b}/{S}_{0}=exp \left[{\left(-b\times DDC\right)}^{a}\right]$$

where b represents the diffusion sensitising factor, S_0,_ and S_b_ represent the signal intensities (SIs) at a b-value of 0 or the b-value indicated by the subscript, respectively. ADC, DDC, and α represent the standard apparent diffusion coefficient, distributed diffusion coefficient, and water molecular diffusion heterogeneity index, respectively [11, 18]. The APTWI parameter was derived from the following formula:

3 $${\text{MTRasym}}\left(3.5{\text{ppm}}\right)=\left[{S}_{sat}\left(-3.5ppm\right)-{S}_{sat}\left(+3.5ppm\right)\right]/{S}_{0}$$

where S_0_ and S_sat_ were the SIs obtained without and with selective saturation, respectively, and MTRasym (3.5 ppm) was the magnetisation transfer ratio asymmetry at 3.5 ppm downfield from the water signal [10]. The regions of interest (ROIs) were manually drawn within the tumour margin layer by layer on the axial T2WI images with reference to the PET/MR fusion image. Areas with cystic degeneration, necrosis, apparent signs and haemorrhage artefacts, and blood vessels were avoided. Subsequently, all completed ROIs were copied to the pseudo colour maps of the MEM-DWI-, SWM-DWI-, and APTWI-derived parameters to calculate the mean values based on the VOI. An attending radiologist and an associate chief radiologist who had 8 and 15 years of experience, respectively, independently performed the above procedures. Both the radiologists were blinded to each other's results and the patient's clinicopathological data.

### Histopathologic evaluation

Within 2 weeks after ^18^F-FDG PET/MRI, surgical resection or biopsy were performed to obtain specimens of all malignant SPLs and 30 benign SPLs. These specimens were sent to our pathology centre for histological analysis [19]. The remaining 11 cases of benign SPLs were followed up for 5–20 weeks to obtain a final diagnosis.

### Statistical analysis

All data were analysed using R (version 3.5.3; R Foundation, Auckland, Zealand) and SPSS (version 15.0; MedCalc Software, Ostend, Belgium). Interobserver consistency for the ^18^F-FDG PET, MEM-DWI, SEM-DWI, and APTWI parameters was assessed using the interclass correlation coefficient (ICC), with interpretations as follows: < 0.40 for poor consistency, 0.40–0.60 for fair consistency, 0.60–0.75 for good consistency, and > 0.75 for excellent consistency [20]. Categorical variables are presented as counts and percentages. Continuous variables are presented as the median and upper and lower quartiles if non-normally distributed and as the mean ± standard deviation if normally distributed. The Mann–Whitney U test, independent samples t-test, and chi-square test were used to compare different variables between the benign and malignant groups. The diagnostic efficacy was evaluated using the area under the receiver operating characteristic curve (AUC), and differences in AUCs were assessed using the DeLong test. The logistic regression (LR) analysis (forward LR method) was used to identify independent predictors and establish a prediction model. Calibration curves and decision curve analysis (DCA) were used for evaluating the prediction model. Statistical significance was set at *P* < 0.05.

## Results

### Basic information

A total of 79 malignant SPLs (11 small cell lung cancer, 16 squamous cell carcinoma, and 52 adenocarcinoma cases) and 41 benign SPLs (eight common inflammation, eight mechanical pneumonia, six tuberculosis, three hamartoma, eight fungal infection, five lung abscess, and three inflammatory pseudotumour cases) were enrolled in this study. Based on the principle of randomisation, 70% of patients in the benign and malignant groups were selected to form a training set (*n* = 84), while the remaining 30% of patients formed a test set (*n* = 36). The patients’ clinical characteristics are summarised in Table 2.

**Table 2:** Summary of characteristics in training and testing sets

Variables	Training set (*n* = 84)	Test set (*n* = 36)	χ^2^ / z / t value	*P* value
Age (year)	62.50 (54.25, 68.00)	56.00 (51.00, 61.75)	- 2.507	0.012 ^a^
Maximum diameter (mm)	29.50 (18.25, 40.75)	25.00 (16.50, 44.50)	- 0.221	0.825 ^a^
Nature of lesion			0.086	0.769
Benign	28 (33.33%)	13 (36.11%)		
Malignant	56 (66.67%)	23 (63.89%)		
Sex			0.007	0.935 ^b^
Male	52 (61.90%)	22 (61.11%)		
Female	32 (38.10%)	14 (38.89%)		
Smoking			1.633	0.201 ^b^
Never	48 (57.14%)	16 (44.44%)		
Always	36 (42.86%)	20 (55.56%)		
Parameters				
SUV_max_	6.76 (4.41, 12.10)	3.66 (2.50, 6.40)	- 3.296	0.001 ^a^
MTV (ml)	7.87 (3.45, 23.12)	8.55 (2.52, 19.06)	- 1.042	0.297 ^a^
TLG (g)	20.73 (5.91, 109.11)	17.65 (3.43, 79.45)	- 1.392	0.164 ^a^
ADC_stand_ (× 10^−3^mm^2^/s)	1.54 ± 0.31	1.43 ± 0.29	- 1.898	0.062 ^c^
DDC (× 10^−3^mm^2^/s)	2.41 (1.69, 3.12)	2.29 (1.54, 2.74)	- 0.988	0.323 ^a^
α	0.53 (0.48, 0.71)	0.62 (0.47, 0.74)	- 0.942	0.346 ^a^
MTRasym(3.5 ppm) (%)	1.64 (0.51, 3.53)	0.88 (0.40, 3.73)	- 0.793	0.428 ^a^

*SUV*_*max*_ Maximum standardized uptake value, *MTV* Metabolic tumor volume, *TLG* Total lesion glycolysis, *ADC* Apparent diffusion coefficient, *DDC* Distributed diffusion coefficient, *α* Diffusion heterogeneity index, *MTRasym* (3.5 ppm) Magnetization transfer ratio asymmetry at 3.5 ppm. A represents the Mann–Whitney U test, b represents the chi-square test and c represents the independent samples t-test

### Consistency test

The SUV_max_, MTV, TLG, ADC, DDC, α, and MTRasym(3.5 ppm) values measured by the 2 radiologists had excellent consistency. The ICC were 0.969 (95% CI: 0.957 ~ 0.979), 0.988 (95% CI: 0.983 ~ 0.992), 0.968 (95% CI: 0.954 ~ 0.978), 0.910 (95% CI: 0.871 ~ 0.938), 0.940 (95% CI: 0.915 ~ 0.958), 0.932 (95% CI: 0.902 ~ 0.953), and 0.897 (95% CI: 0.852 ~ 0.928), respectively. The average results were used for the ultimate analysis.

### Parameter comparison

**Fig. 2:**
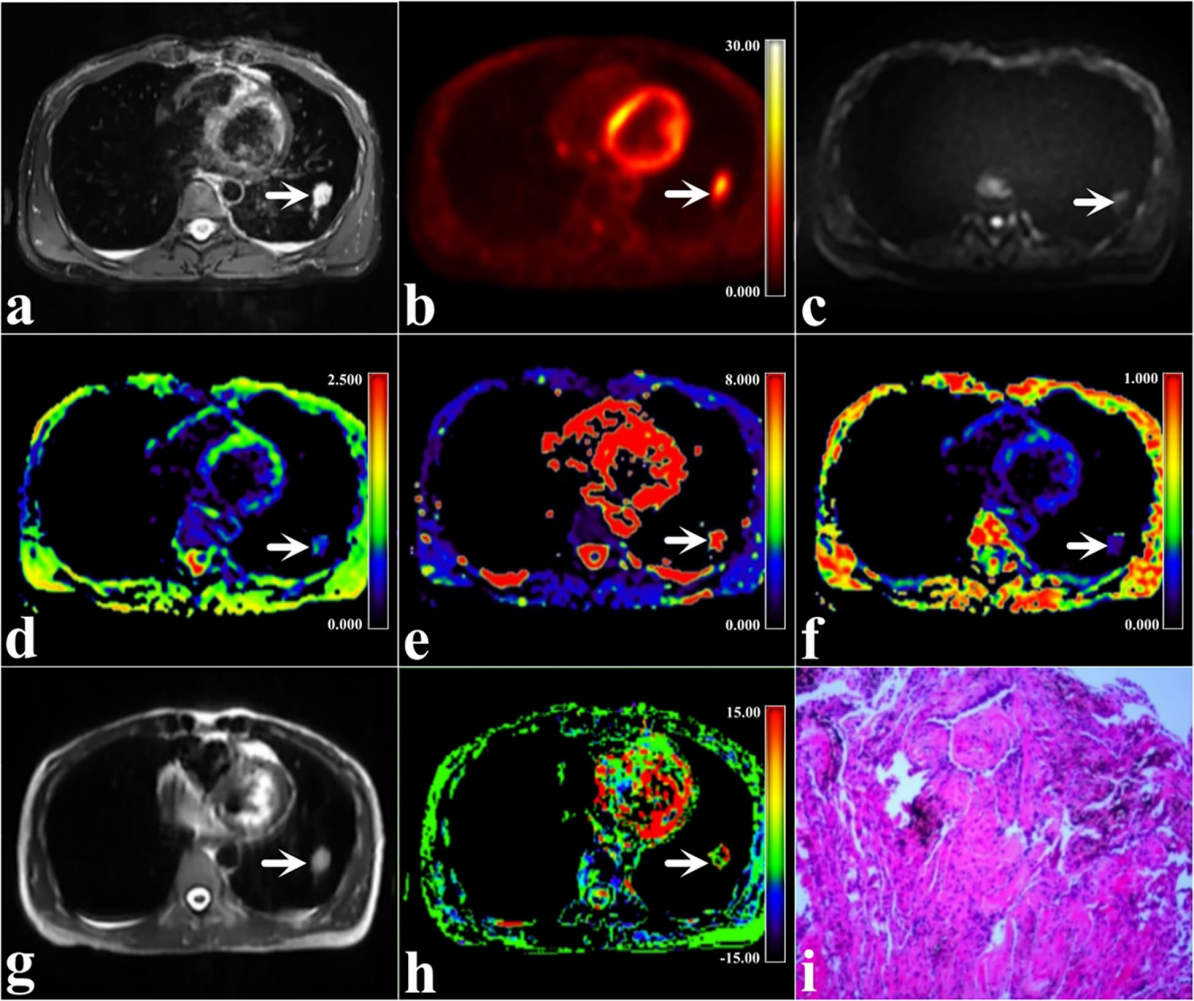
A 35-year-old woman with benign SPLs in the lower lobe of the left lung (arrowheads, size 15 mm × 25 mm × 21 mm, fibrous tissue hyperplasia with chronic inflammation). **a** Map of T2WI; **b** Map of ^18^F-FDG PET; (**c)** Map of MEM-DWI (b = 600 s/mm.^2^); (**d)** Pseudo colored map of ADC; (**e)** Pseudo colored map of DDC; (**f)** Pseudo colored map of α; (**g**) Map of APTWI; (**h**) Pseudo colored map of MTRasym(3.5 ppm); and (**i**) Pathological images (H&E staining,100 ×)

**Fig. 3:**
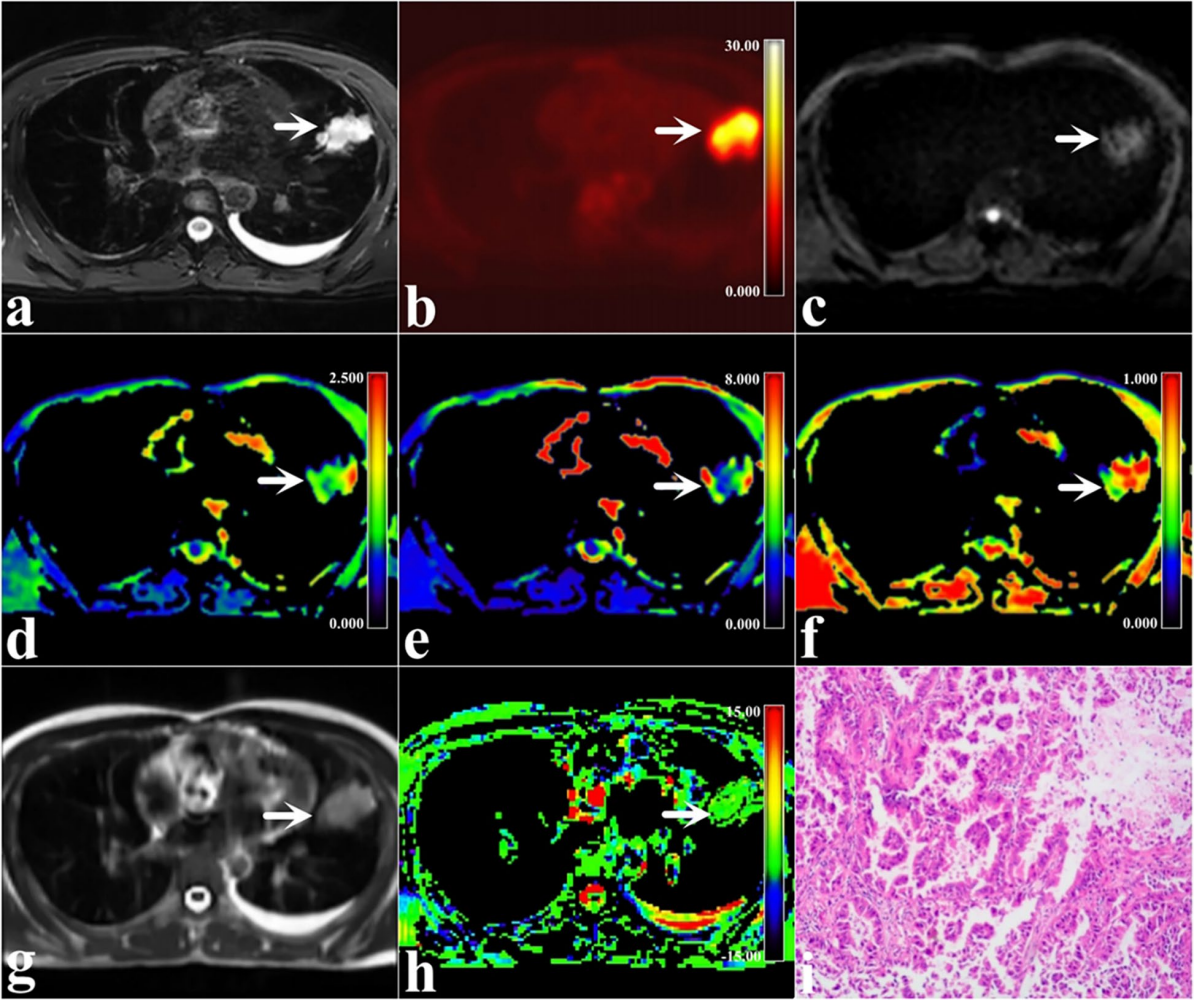
A 35-year-old man with malignant SPLs in the high lobe of the left lung (arrowheads, size 50 mm × 50 mm × 40 mm, mucinous adenocarcinoma). **a** Map of T2WI; (**b)** Map of ^18^F-FDG PET; (**c)** Map of MEM-DWI (b = 600 s/mm.^2^); (**d)** Pseudo colored map of ADC; (**e)** Pseudo colored map of DDC; (**f)** Pseudo colored map of α; (**g)** Map of APTWI; (**h)** Pseudo colored map of MTRasym(3.5 ppm); and (**i)** Pathological images (H&E staining,100 ×)

SUV_max_, MTV, TLG, α, and MTRasym(3.5 ppm) values were significantly lower and ADC, DDC values were significantly higher in benign SPL than malignant SPL (*P* < 0.001, < 0.001, < 0.001, = 0.004, < 0.001, < 0.001, and < 0.001, respectively, Table 3, Figs. 2 and 3).

**Table 3:** Comparison of different characteristics between benign and malignant group in the training set

Variables	Benign group (*n* = 84)	Malignant group (*n* = 36)	χ^2^ / z / t value	*P* value
Age (year)	57.54 ± 7.98	61.59 ± 11.96	- 1.845	0.069 ^a^
Maximum diameter (mm)	16.50 (9.25, 31.00)	31.50 (24.00, 46.25)	- 3.551	< 0.001 ^b^
Sex			0.404	0.525 ^c^
Male	16 (57.14%)	36 (64.29%)		
Female	12 (42.86%)	20 (35.71%)		
Smoking			0.219	0.640 ^c^
Never	17 (60.71%)	31 (55.36%)		
Always	11 (39.29%)	25 (44.64%)		
Parameters				
SUV_max_	4.38 (2.78, 5.44)	9.63 (6.23, 13.23)	- 5.257	< 0.001 ^b^
MTV (ml)	4.05 (2.33, 6.78)	14.46 (4.77, 38.82)	- 3.981	< 0.001 ^b^
TLG (g)	6.29 (1.86, 12.96)	62.16 (11.33, 155.71)	- 4.602	< 0.001 ^b^
ADC (× 10^−3^mm^2^/s)	1.79 ± 0.19	1.42 ± 0.28	7.266	< 0.001 ^a^
DDC (× 10^−3^mm^2^/s)	3.00 (2.78, 3.32)	1.93 (1.57, 2.60)	- 4.261	< 0.001 ^b^
α	0.48 (0.42, 0.52)	0.63 (0.50, 0.78)	- 4.251	< 0.001 ^b^
MTRasym(3.5 ppm) (%)	1.07 (0.35, 1.75)	2.58 (0.62, 3.87)	- 2.847	0.004 ^b^

*SUV*_*max*_ Maximum standardized uptake value, *MTV* Metabolic tumor volume, *TLG* Total lesion glycolysis, *ADC* Apparent diffusion coefficient, *DDC* Distributed diffusion coefficient, *α* Diffusion heterogeneity index, *MTRasym* (3.5 ppm) Magnetization transfer ratio asymmetry at 3.5 ppm. A represents the Mann–Whitney U test, b represents the chi-square test and c represents the independent samples t-test

### Regression analyses

The potential risk-related factors such as age, maximum diameter, sex, smoking, SUV_max_, MTV, TLG, ADC, DDC, α, and MTRasym(3.5 ppm) were all enrolled in regression analysis. Univariate analysis demonstrated that maximum diameter, SUV_max_, MTV, TLG, ADC, DDC, α, and MTRasym(3.5 ppm) were all risk predictors (P all < 0.05), while multivariate analysis showed that only SUV_max_, ADC, and MTRasym(3.5 ppm) were independent predictors (*P* = 0.001, 0.001, and 0.024, respectively, Table 4).

**Table 4:** Univariate and multivariate analyses

Variables	Univariate Analyses		Multivariate Analyses	
	OR (95% CI)	*P*-value	OR (95% CI)	*P*-value
Age (year)	1.447 (0.914 ~ 2.290)	0.115	/	**/**
Sex	1.158 (0.736 ~ 1.821)	0.526	/	**/**
Smoking	0.896 (0.566 ~ 1.419)	0.640	/	**/**
Maximum diameter (mm)	2.279 (1.224 ~ 4.245)	0.009	/	**/**
SUV_max_	9.892 (3.253 ~ 30.082)	< 0.001	61.636 (5.636 ~ 674.116)	0.001
MTV (ml)	4.330 (1.184 ~ 15.832)	0.027	/	**/**
TLG (g)	31.119 (2.786 ~ 347.591)	0.005	/	**/**
ADC (× 10^−3^mm^2^/s)	0.128 (0.050 ~ 0.329)	< 0.001	0.017 (0.002 ~ 0.181)	0.001
DDC (× 10^−3^mm^2^/s)	0.406 (0.226 ~ 0.728)	0.002	/	**/**
α	3.696 (1.816 ~ 7.522)	< 0.001	/	**/**
MTRasym(3.5 ppm) (%)	2.085 (1.142 ~ 3.804)	0.017	11.178 (1.378 ~ 90.661)	0.024

Both univariate and multivariate analyses were conducted using the forward LR method

*SUV*_*max*_ Maximum standardized uptake value, *MTV* Metabolic tumor volume, *TLG* Total lesion glycolysis, *ADC* Apparent diffusion coefficient, *DDC* Distributed diffusion coefficient, *α* Diffusion heterogeneity index, *MTRasym* (3.5 ppm) Magnetization transfer ratio asymmetry at 3.5 ppm, *OR* Odds ratio; *OR for per 1 standard deviation, *CI* Confidence interval

### Diagnostic performance

**Fig. 4:**
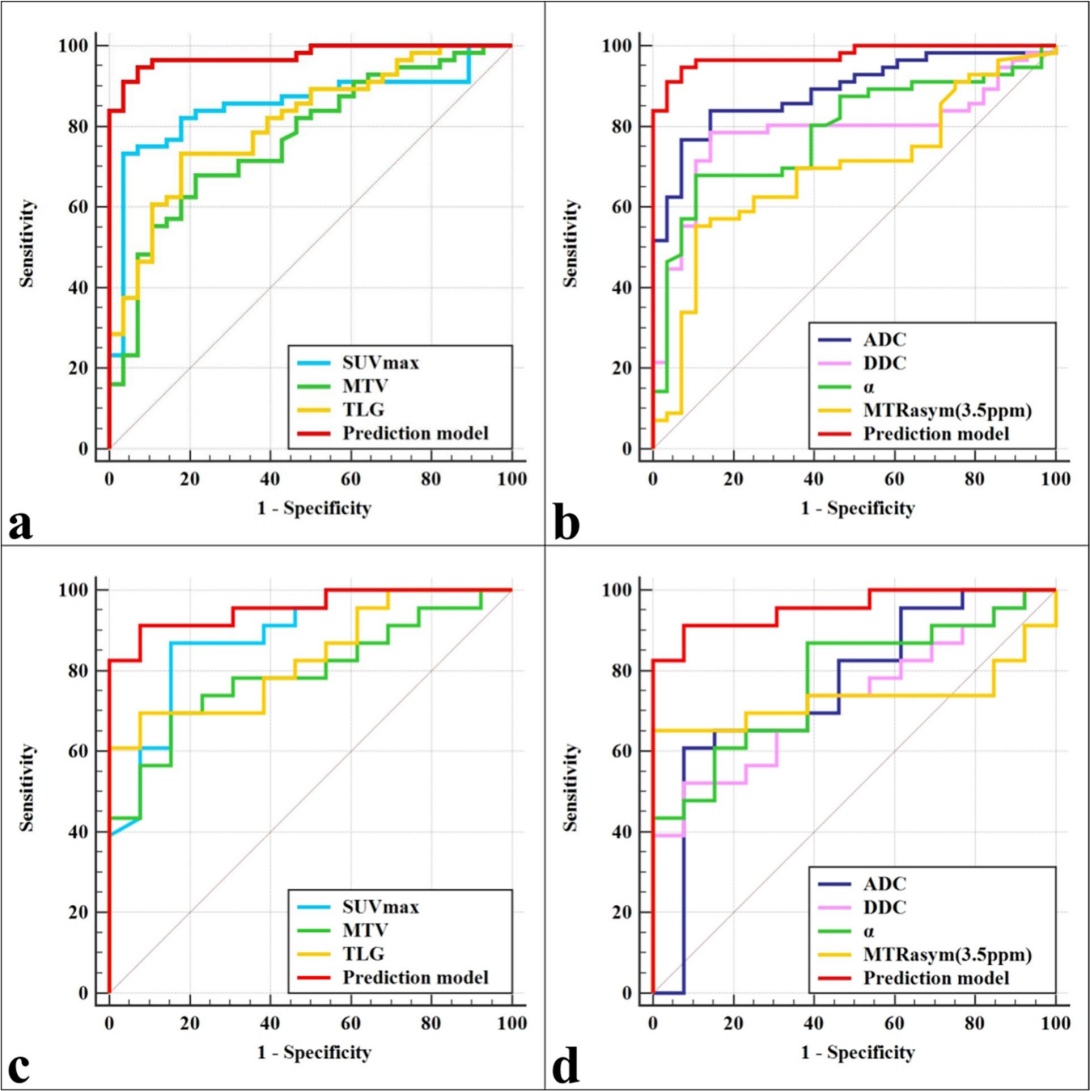
The area under receiver-operator characteristic (ROC) curves of different parameters and the prediction model. **a**, **b** ROC curve of the training set. **c**, **d** ROC curve of the test set

Within the training set, the prediction model based on these independent predictors demonstrated optimal diagnostic efficacy (AUC, 0.976; sensitivity, 94.64%; specificity, 92.86%), surpassing ADC, SUV_max_, TLG, DDC, α, MTV, and MTRasym (3.5 ppm) with statistical significance (AUC = 0.888, 0.853, 0.809, 0.786, 0.786, 0.768, and 0.691, Z = 2.761, 3.080, 3.653, 3.844, 3.773, 4.030, and 4.938, respectively, *P* = 0.006, 0.002, < 0.001, < 0.001, < 0.001, < 0.001, and < 0.001, respectively). Similarly, within the test set, the prediction model exhibited optimal diagnostic efficacy (AUC, 0.957; sensitivity, 91.30%; specificity, 92.31%, Table 5, Fig. 4), outperforming TLG, MTV, α, ADC, MTRasym (3.5 ppm), and DDC with statistical significance (AUC = 0.833, 0.793, 0.776, 0.756, 0.732, and 0.729, Z = 2.317, 2.337, 2.514, 2.263, 2.562, and 2.697, respectively, *P* = 0.021, 0.020, 0.012, 0.024, 0.010, and 0.007, respectively).

**Table 5:** Predictive performance for identifying benign and malignant SPL

Parameters	AUC (95% CI)	*P*-value	Cutoff	Sensitivity	Specificity	Comparison with a combined diagnosis
Training set						
SUV_max_	0.853 (0.759 ~ 0.921)	< 0.001	6.690	73.21%	96.43%	Z = 3.080, *P* = 0.002
MTV (ml)	0.768 (0.663 ~ 0.853)	< 0.001	6.930	67.86%	78.57%	Z = 4.030, *P* < 0.001
TLG (g)	0.809 (0.709 ~ 0.887)	< 0.001	16.653	73.21%	82.14%	Z = 3.653, *P* < 0.001
ADC (× 10^−3^mm^2^/s)	0.888 (0.800 ~ 0.946)	< 0.001	1.573	76.79%	92.86%	Z = 2.761, *P* = 0.006
DDC (× 10^−3^mm^2^/s)	0.786 (0.683 ~ 0.868)	< 0.001	2.721	78.57%	85.71%	Z = 3.844, *P* < 0.001
α	0.786 (0.683 ~ 0.868)	< 0.001	0.534	67.86%	89.29%	Z = 3.773, *P* < 0.001
MTRasym(3.5 ppm) (%)	0.691 (0.581 ~ 0.788)	0.002	2.225	55.36%	89.29%	Z = 4.938, *P* < 0.001
Prediction model	0.976 (0.916—0.997)	< 0.001	/	94.64%	92.86%	/
Test set						
SUV_max_	0.885 (0.734 ~ 0.967)	< 0.001	3.330	86.96%	84.62%	Z = 1.741, *P* = 0.081
MTV (ml)	0.793 (0.625 ~ 0.909)	< 0.001	8.395	69.57%	84.62%	Z = 2.337, *P* = 0.020
TLG (g)	0.833 (0.671 ~ 0.936)	< 0.001	17.924	69.57%	92.31%	Z = 2.317, *P* = 0.021
ADC (× 10^−3^mm^2^/s)	0.756 (0.584 ~ 0.883)	0.004	1.324	60.87%	92.31%	Z = 2.263, *P* = 0.024
DDC (× 10^−3^mm^2^/s)	0.729 (0.555 ~ 0.863)	0.006	1.824	52.17%	92.31%	Z = 2.697, *P* = 0.007
α	0.776 (0.606 ~ 0.898)	< 0.001	0.485	86.96%	61.54%	Z = 2.514, *P* = 0.012
MTRasym(3.5 ppm) (%)	0.732 (0.559 ~ 0.866)	0.007	1.880	65.22%	100.00%	Z = 2.562, *P* = 0.010
Prediction model	0.957 (0.831—0.997)	< 0.001	/	91.30%	92.31%	/

*SPL* solitary pulmonary lesion, *SUV*_*max*_ Maximum standardized uptake value, *MTV* Metabolic tumor volume, *TLG* Total lesion glycolysis, *ADC* Apparent diffusion coefficient, *DDC* Distributed diffusion coefficient, *α* diffusion heterogeneity index, *MTRasym*(3.5 ppm) magnetization transfer ratio asymmetry at 3.5 ppm. The prediction model represents SUV_max_. + ADC + MTRasym (3.5 ppm)

### Validation

**Fig. 5:**
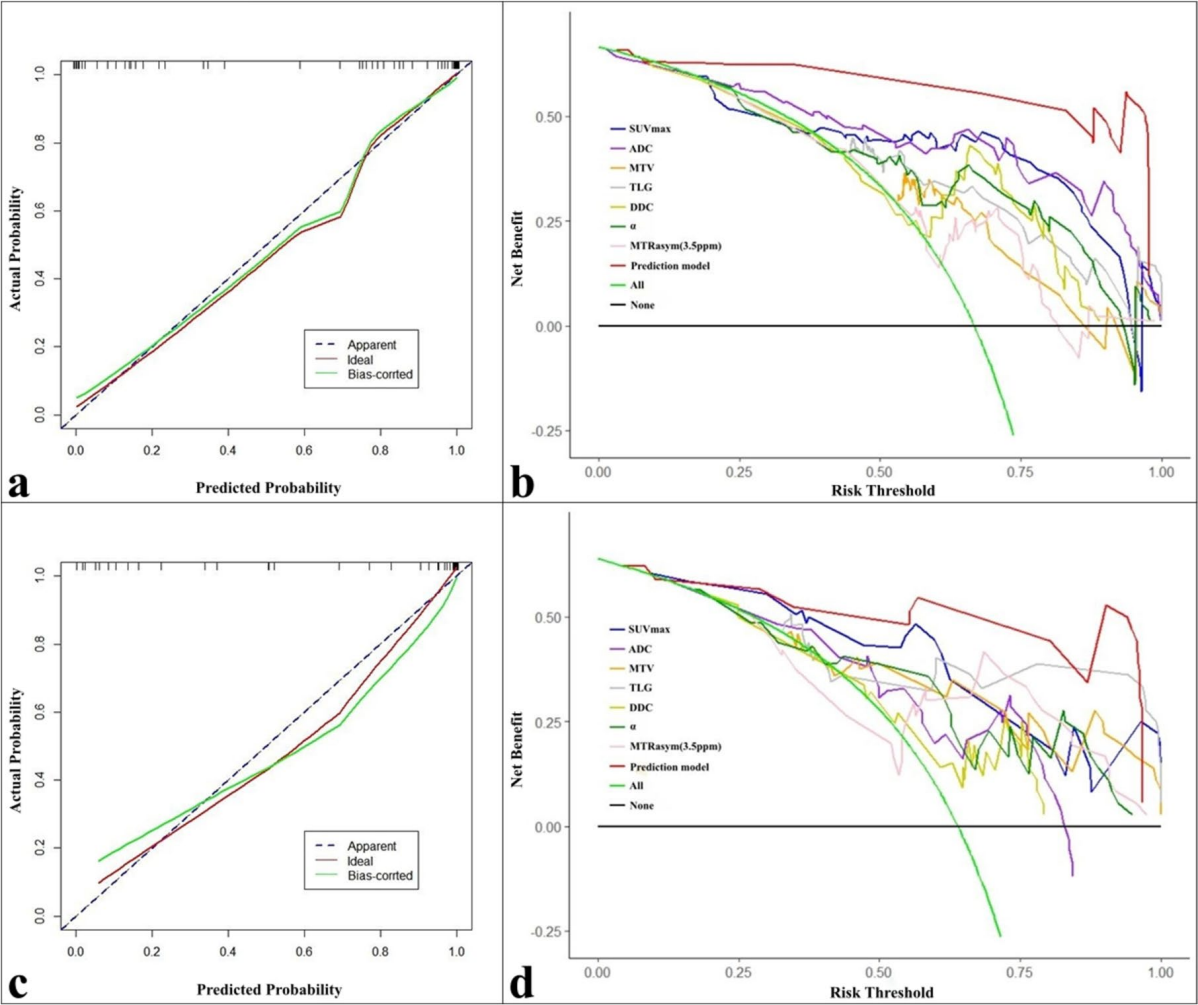
Calibration curves and decision curve analysis (DCA) curves. **a**, **b** Calibration curve and DCA of the training set. **c**, **d** Calibration curve curve and DCA of the test set

The calibration curves and DCA revealed that the prediction model exhibited not only good consistency but also provided greater clinical benefits to the relevant patients compared with any single parameter, as evident in the training and test sets (Fig. 5).

## Discussion

Currently, ^18^F-FDG PET is widely employed as a molecular imaging technique in clinical practice. It provides valuable information regarding the metabolism of the target tissue, with derived parameters such as SUV_max_, TLG, and MTV commonly used to quantify glucose metabolism. SUV_max_ primarily reflects the highest levels of glucose metabolism within the target region, while TLG and MTV reflect the overall glucose metabolism in the target region [21]. Erdoğan et al. comprehensively evaluated ^18^F-FDG PET data from 113 patients with lung lesions and observed that SUV_max_, TLG, and MTV all played a positive role in distinguishing between benign and malignant lung lesions [22]. In a meta-analysis conducted by Li et al., the role of ^18^F-FDG PET in evaluating lung lesions was assessed. The findings demonstrated that although there was some overlap in ^18^F-FDG uptake between benign and malignant lesions, ^18^F-FDG PET remained a reliable diagnostic tool [23]. In this study, malignant SPLs exhibited elevated metabolic activity and higher ^18^F-FDG transport and uptake compared with benign SPLs. Consequently, there were significant increases in SUV_max_, MTV, and TLG in the malignant group. Furthermore, the LR analysis confirmed that SUV_max_ served as an independent predictor for discriminating between benign and malignant SPLs, which is consistent with the above findings, further suggesting that ^18^F-FDG PET could help differentiate between benign and malignant SPLs.

The MEM-DWI represents the first DWI method to be used in clinical practice and assumes a uniform Gaussian distribution for the diffusion motion of water molecules in biological tissues. It uses the quantitative parameter ADC to capture variations in water molecule diffusion motion [24]. The value of ADC has been extensively investigated for its potential in differentiating between benign and malignant lung lesions. Numerous studies have consistently reported significantly lower ADC values in malignant lesions compared with benign lesions, primarily attributed to the increased cell proliferation, dense tissue structure, and more restricted diffusion of water molecules within the malignant lesions [25]. In our study, the ADC values in malignant SPLs were significantly lower compared with those in benign SPLs. Furthermore, ADC emerged as an independent predictor for differentiating between benign and malignant SPLs, which is consistent with the previous studies and further demonstrates the role of MEM-DWI in differentiating between benign and malignant SPLs.

In contrast to MEM-DWI, SEM-DWI assumes that the movement of water molecules in biological tissues occurs within a non-homogeneous environment. This approach yields two quantitative parameters, namely DDC, which reflects the distributed diffusion of water molecules, and α, which reflects tissue heterogeneity [11]. Similar to ADC, DDC values are primarily influenced by the tightness of the tissue structure, In this study, patients with malignant SPLs, characterised by increased cell proliferation and tighter structure, exhibited significantly lower DDC values compared to those with benign SPLs. On the other hand, the magnitude of α is closely associated with tissue heterogeneity. Previous studies on endometrial carcinoma [26], breast cancer [27], and renal cancer [28] have demonstrated that more malignant lesions generally exhibit significant tissue heterogeneity due to factors such as necrosis, haemorrhage, and cellular heterogeneity, resulting in reduced α values. Surprisingly, the findings of this study revealed an opposite trend, wherein the α value was increased in malignant SPLs compared with benign SPLs. It is speculated that this discrepancy may be attributed to the specific structure of the lung tissue. In benign SPLs, although there is relatively less necrosis, haemorrhage, and cellular heterogeneity, it contains more normal lung tissues such as alveoli and fine bronchi. These elements could contribute to increased tissue heterogeneity to some extent. Conversely, in malignant SPLs, despite the presence of more pronounced necrosis, haemorrhage, and cellular heterogeneity, tissues such as alveoli and fine bronchi are often replaced by cancer cells, thereby reducing the heterogeneity [29]. Nevertheless, the accuracy of this inference should be validated, given the limited application of SEM-DWI in lung lesions.

APTWI, a quantitative MRI sequence based on CEST, uses proton exchange to transfer variations in tissue-mobile protein/peptide concentrations to water molecules. It calculates the quantitative parameter MTRasym (3.5 ppm) to reflect changes in mobile protein and peptide concentrations within biological tissues [10]. This study aimed to compare the differences in MTRasym (3.5 ppm) values between patients with benign and malignant SPLs. The results indicated that patients in the malignant SPL group exhibited significantly higher MTRasym (3.5 ppm) values than those in the benign SPLs group, consistent with the findings of Ohno et al. [13, 14]. These findings further suggest the potential of APTWI in aiding the qualitative diagnosis of SPLs. One possible explanation for this outcome is that patients with benign SPLs tend to have more robust cell proliferation and a higher presence of necrotic and haemorrhagic components. Consequently, there is an increased concentration of mobile protein/peptide within the tissue, resulting in increased MTRasym (3.5 ppm) values [30–32].

Due to the inherent tissue heterogeneity in tumourous lesions, relying on a single parameter for a comprehensive and accurate assessment can be challenging [33]. Previous studies have highlighted the advantages of using multi-parameter combination diagnosis based on multiple quantitative or semi-quantitative parameters, particularly multiple independent predictors, in improving the diagnosis and evaluation of tumours compared with relying on single parameters [34, 35]. Accordingly, the study employed univariate and multivariate LR analysis to identify independent predictors (SUV_max_, MTRasym [3.5 ppm], and ADC) for differentiating between benign and malignant SPLs among various clinical factors and quantitative and semi-quantitative parameters. Based on these predictors, a corresponding prediction model was developed. The results demonstrated that the prediction model not only exhibited varying degrees of improved diagnostic efficacy compared with individual parameters but also provided reliable benefits to the patients, as observed in both the training and test sets. These findings suggest that the combination of multiple parameters might more and accurately reflect the characteristics of the lesion, emphasising the importance of using as many imaging methods as possible to assess patients whenever feasible.

While this study yielded encouraging results, it is important to acknowledge several limitations. First, the study was conducted at a single institution, and although a training and test set were employed, the sample size remained relatively small. Additionally, the external validation of these findings across multiple institutions was not performed, which might affect the reliability of the results to some extent. Second, the exclusion of some microscopic lesions, particularly benign SPLs, from ^18^F-FDG PET/MRI scans due to poor display could limit the applicability of the study. Third, respiratory and cardiovascular pulsation artefacts in the lungs can be significant, despite attempts to mitigate their effects through various techniques. These artefacts might still influence the stability of the various quantitative/semi-quantitative parameters. Fourthly, the present study has not explored the value of APTWI, SEM-DWI, and ^18^F-FDG PET in the assessment of different histopathological features of SPL such as Ki-67, grade, etc., which may have led to an inadequate study. Future studies should aim to expand the sample size, conduct multi-center studies, asses a wider range of histopathological features, and explore technologies that can reduce artefacts, such as cardiovascular gating, rapid scanning and electrocardiogram triggering, to enhance imaging quality and obtain more stable and reliable experimental results.

## Conclusion

Multiparametric PET/MRI based on ^18^ F-FDG PET, MEM-DWI, SEM-DWI, and APTWI can effectively evaluate the characteristics of SPLs. The prediction model comprising SUV_max_, ADC, and MTRasym (3.5 ppm) demonstrated superior diagnostic efficacy compared with individual parameters. It holds promise as a reliable imaging marker for differentiating between benign and malignant SPLs.

## Data Availability

The datasets during and/or analysed during the current study available from the corresponding author on reasonable request.

## Abbreviations

APTWI: Amide proton transfer-weighted imaging
^18^F-FDG PET: ^18^F-Fluorodeoxyglucose positron emission tomography
SPLs: Solitary pulmonary lesions
PFS: Progression-free survival
ADC: Apparent diffusion coefficient
DDC: Distributed diffusion coefficient
α: Water molecular diffusion heterogeneity index
MTRasym (3.5 ppm): Magnetization transfer ratio asymmetry at 3.5 ppm
SUV_max_: Maximum standardized uptake value
MTV: Metabolic tumor volume
TLG: Total lesion glycolysis
